# Intracranial Pressure and Its Relationship to Glaucoma: Current Understanding and Future Directions

**Published:** 2015

**Authors:** Uttio ROY CHOWDHURY, Michael P. FAUTSCH

**Affiliations:** 1 Department of Ophthalmology, Mayo Clinic, Rochester, MN, United States

**Keywords:** Intracranial Pressure, Cerebrospinal Fluid Pressure, Glaucoma, Lamina Cribrosa, Intraocular Pressure

## Abstract

Retrospective and prospective studies looking at the role of cerebrospinal fluid pressure (CSFP)/intracranial pressure (ICP) have stimulated new theories and hypotheses regarding the underlying causal events for glaucoma. Most recently, studies supporting a low CSFP/ICP as a risk factor for glaucoma have been published. This review summarizes the current understanding of CSFP/ICP and its potential role in the pathogenicity of the disease.

## INTRODUCTION

Glaucoma is defined as a collection of diseases that causes progressive degeneration of optic neurons. Characteristic damage to axonal components of the optic nerve head and the surrounding neuroretinal rim, also takes place (1, 2). Despite advances in clinical management strategies, glaucoma continues to be the leading cause of irreversible blindness worldwide (3). The most common form of the disease is primary open-angle glaucoma (POAG), and it usually manifests with an elevated intraocular pressure (IOP). Elevated IOP is a well-recognized risk factor for glaucoma (4) and is also a causal factor for optic neuronal damage. As a result, all current treatment modalities for glaucoma are geared toward the reduction of IOP (5). The exact sequence of events that cause glaucomatous optic nerve damage after an elevated IOP remains unclear (6, 7). In addition to this, a large number of patients who suffer from normal tension glaucoma (NTG) develop glaucomatous optic nerve damage despite having an IOP below 22 mmHg (6). In fact, depending on race and ethnicity, NTG may be the most predominant form of glaucoma. This is particularly true in Asian populations where the majority of POAG belong to the NTG subtype (52-92%) (8). NTG proportions are comparatively lower in Caucasian (30-38.9%) and African populations (57.1%) (8). Furthermore, there is a distinct group of patients with high IOP (ocular hypertension), who never develop glaucoma (7, 9). Many glaucoma patients whose IOP is maintained at a safe target level, continue to lose optic neurons with time, therefore requiring a continuous reassessment of their target IOP (4, 10, 11). In light of these evidences, there clearly appears to be risk factors other than IOP that are involved in the progression of the glaucoma pathology.

Probability of developing glaucoma is directly linked to the number and strength of risk factors (7). Other than elevated IOP, important risk factors include race, ethnicity and age. Likelihood of glaucoma was found to be high when there was a familial predisposition to the disease. Clinically, an increased or asymmetric cup to disk ratio and findings of disk hemorrhage were found to correlate strongly with subsequent development of glaucoma (4, 7, 12) as did a long-term treatment with topical corticosteroids (13). Recently a number of studies have shown that cerebrospinal fluid pressure (CSFP) or intracranial pressure (ICP) is a significant risk factor for glaucoma. Retrospective and prospective studies looking at the role of CSFP/ICP have stimulated new theories and hypotheses regarding the underlying causal events for glaucoma. In this review, we examine the current literature and studies regarding CSFP/ICP with reference to glaucoma and evaluate how findings from these reports can help further our knowledge in understanding the pathogenicity of the disease.

## CEREBROSPINAL FLUID PRESSURE/INTRACRANIAL PRESSURE

Cerebrospinal fluid (CSF) is a continually generated specialized liquid secreted mostly (70-80%) by the choroid plexus in the lateral, third and fourth ventricles (14). The cerebral capillary wall also secretes a CSF like fluid (15), but it is not as efficient as the choroid plexus. Nascent CSF is much enriched by active secretion of the choroidal epithelium (16). The rate of CSF secretion is tightly regulated by multiple choroidal and extra choroidal mechanisms to maintain a stable CSFP/ICP (14). When measured by lumbar puncture, CSFP directly correlates with ICP and the retrolaminar pressure measured in the lateral decubitus position. As a result, ICP and CSFP have been used interchangeably in the medical literature and clinical practice (17-20). It should be remembered that while ICP indicates the pressure inside the cranium, CSFP is the more general term referring to the pressure throughout the neuroaxial system (18). In connection to glaucoma, ICP or the CSFP of the cranium is more relevant and henceforth for the purpose of this review, the pressure created by CSF will be referred to as ICP.

## PRESSURE COMPONENTS AFFECTING THE OPTIC NERVE: AN ANATOMICAL PERSPECTIVE

The optic nerve is a myelinated tract of approximately 1.2 million axons that start from the retinal ganglion cells and end after traveling approximately 50 mm to the optic chiasm. The optic tract is the posterior projection of the optic nerve beyond the optic chiasma until its termination (21). Of the various portions of the optic nerve, the intraocular part or the optic disc is clinically most relevant to the pathophysiology of glaucoma. This is the area where the characteristic optic nerve cupping is observed. The intraocular portion of the optic nerve has three distinct anatomical zones: a) the retinal or prelaminar zone; b) the choroidal or laminar zone; and c) the scleral or retrolaminar/retrobulbar zone. As the retinal ganglion axons make an orthogonal turn from the nerve fiber layer and pass through the lamina cribrosa, there is a sudden change in the surrounding pressure. The axons move from a higher IOP to a comparatively lower retrobulbar pressure. After emerging out of the lamina cribrosa and throughout its whole course, the optic nerve is surrounded and bathed by CSF, running through the sub arachnoid space and generating the so-called retrobulbar pressure which in essence is the same as ICP (20). Therefore, over the span of a few millimeters, the optic nerve experiences two different pressure components - the higher IOP and the lower ICP. These two pressure components are separated by the lamina cribrosa which is a thin collagenous support of the optic nerve and is basically a perforated region of the posterior sclera that allows the retinal axons to pass into the optic nerve (20, 22, 23).

## THE LAMINA CRIBROSA AND THE TRANSLAMINAR PRESSURE GRADIENT

Due to changes in pressure, the lamina cribrosa deforms posteriorly during glaucoma and anteriorly during papilledema, pseudotumor cerebri, and ocular hypotony; all diseases associated with loss of axons and retinal neurons (20, 24). Because of this, the lamina cribrosa is considered the anatomic landmark of interest in glaucoma and other diseases where IOP and ICP are important contributory factors (25). As mentioned before, at any given time the lamina cribrosa is under the influence of two separate but possibly interdependent pressures – the posteriorly acting IOP and the anteriorly acting ICP. The difference in these two pressures creates the translaminar pressure gradient (TLPG). In addition to IOP and ICP, the thickness of the lamina cribrosa also plays a significant role in determining this pressure gradient (17, 26, 27). Therefore, the TLPG may be defined as the difference between IOP and ICP per unit thickness of the lamina cribrosa [(IOP-ICP)/thickness of the lamina cribrosa] (17). The ability of the lamina cribrosa to withstand this pressure gradient also depends on the surrounding extracellular matrix and the peripheral scleral tension (28). It should be noted that the distribution of the pressure gradient might not be uniform even in the presence of a constant IOP. According to the Law of LaPlace, the tension on the walls of the structure is dependent on the IOP and radius of curvature divided by 2 times the thickness of the wall. Therefore, the inherent heterogeneity of the lamina cribrosa also plays a significant role in determining the distribution of the pressure gradient (25). Another factor affecting the TLPG is the pulsatile movements of the lamina cribrosa in a coronary-sagittal plane (29). Although the physiologic significance of these changes is unclear, they may have a role in facilitating orthograde and retrograde axoplasmic flow (29, 30). Nevertheless, the integrity and resilience of the lamina cribrosa in maintaining its shape is extremely important for protecting the health of the structures that pass through it – mainly the axonal components of the retinal ganglion cells as well as the arterial and venous vessels (28, 31). In fact, posterior cupping of the lamina cribrosa is one of the hallmark pathologic manifestations of glaucoma and occurs quite early in the disease (32).

**Figure 1:**
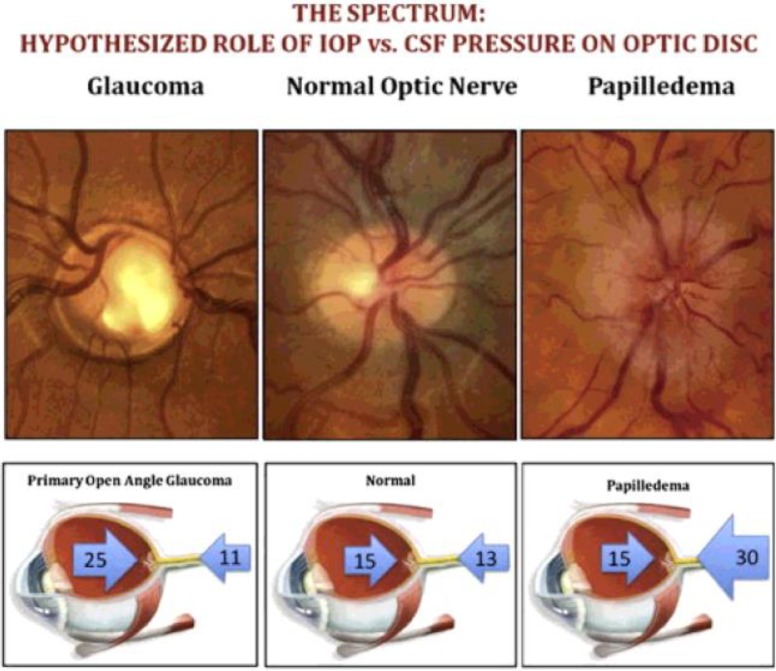
Schematic representation of the CSF theory.

The thickness of a normal lamina cribrosa is around 450 µm with a calculated pressure gradient of about 1 mmHg per 100 µm (17, 33). This is one of the steepest pressure gradients to which a nerve is exposed in the human body (17). It is widely accepted that pressure gradients across nervous tissues can alter axonal transport (23). In peripheral nerve compression studies, it has been shown that the pressure gradient and not the absolute pressure increase (as high as 5 atmospheres) were responsible for blocking orthograde axonal transport (34-36). Given the fact that lamina cribrosa is under the continuous influence of two separate pressure components, it is reasonable to believe that significant pathological deformities of the lamina cribrosa can originate from changes in either pressure components. Indeed, recent studies clearly show that ICP is as responsible for causing neuronal damage at the optic nerve head as elevated IOP (18, 20, 27, 37-39). To acknowledge the contribution of ICP in glaucomatous optic nerve damage, Fleishman and Berdahl have postulated the CSF theory of glaucoma (25). According to this theory, a net balancing force arising out of the difference between IOP and ICP determines the TLPG. The TLPG is affected by changes in either IOP and/or ICP. For example, a reduction in ICP will have the same effect as an increase in IOP (Fig. 1). This gradient is involved in all kinds of laminar movement and consequently all pathologic cupping (as seen in glaucoma) or swelling (as seen in papilledema). A large body of evidence now exists to show that ICP is indeed a formidable risk factor for the development of glaucoma.

The homeostatic translaminar pressure difference is maintained by the interaction of two pressure components – the higher IOP and the lower ICP. Changes in either parameter can cause disruption of the pressure gradient and lead to optic neuronal damage. Reprinted from Fleishman et al. (8)

## EVIDENCE IN FAVOR OF ICP AS A RISK FACTOR FOR GLAUCOMA

More than 90 years ago, Szymanski and Wladyczko proposed that low ICP may be a factor in the development of glaucoma (40). Since then, this concept has fallen in and out of favor over the following years. In 1976, Volkov postulated that the retrolaminar tissue pressure is affected by ICP particularly because the optic nerve immediately beyond the lamina cribrosa is surrounded by CSF (41). Within a few years of this report Yablonski et al. published two abstracts that suggested glaucoma like changes in cat eyes when intracranial pressure was lowered below atmospheric pressure. These changes included axonal swelling, increased cup to disk ratio and cupping of the lamina cribrosa. They also were able to counteract this effect of low ICP by lowering the IOP at the same time. (Yablonski ME, et al. IOVS 1978;17: ARVO Abstract 6; Yablonski ME, et al. IOVS 1979;18: ARVO Abstract 8) Although these data were the first experimental proof of the involvement of ICP in glaucomatous pathology, the authors never published their results formally, and it is hard to comment on the merits of the studies. A few years later, glaucoma like cupping in several patients with normal IOP but suffering from compression of the intracranial optic nerve owing to tumor or other lesions were reported (42, 43). In their case report, Kalenak et al. mentioned a highly unusual unilateral NTG due to tumorigenic blockage of the optic nerve in one eye (42). It should be mentioned here that glaucoma owing to nerve compression by a pituitary tumor (Rathke’s cleft cyst) has also been reported in later years and intracranial compressive lesions are often taken into account during diagnosis of normal tension glaucoma (NTG) (44-46). Although the authors did not report the ICP of these patients, it is possible that due to compression caused by the tumor, pressure in the optic nerve subarachnoid space fell below the ICP and induced glaucoma like pathologies. Therefore, though unrecognized, these may be the first clinical studies showing the involvement of ICP in causing glaucoma like changes in the eye. Shortly thereafter, in a series of elegant studies, Morgan et al. provided solid experimental evidence of the importance of ICP in maintaining the translaminar pressure gradient and subsequently the health of the surrounding optic neuronal tissues (20). They were able to calculate the TLPG in a dog model and determined that both ICP and IOP are responsible for creating a homeostatic TLPG. The authors further proved that ICP in the brain is the same as the retrobulbar pressure owing to an anatomic and hydrostatic continuity of the fluid-filled tracts (20). At the same time, Shin et al. independently commented on the importance of the “retrolaminar intraoptic nerve pressure” on displacement of the lamina cribrosa, from their observations of adult glaucoma patients (47-50).

**Figure 2:**
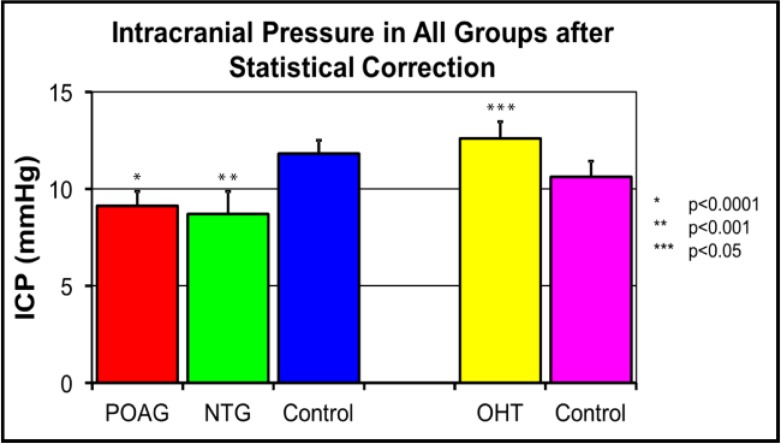
Statistically corrected intracranial pressure across various groups of glaucoma and ocular hypertension (OHT), in comparison to respective controls.ICP was significantly lower in POAG and NTG groups and higher in OHT patients. Reprinted from Berdahl et al. (18).

Intracranial pressure with reference to glaucoma received much attention during the late 2000s, owing to some well-designed retrospective studies by Drs. Fautsch, Johnson, Allingham, Berdahl and Fleischman. To look for possible associations between ICP and glaucoma the authors used an integrated and comprehensive multispecialty database of the Mayo Clinic. They analyzed the medical history of thousands of patients who underwent lumbar puncture for measurement of ICP. Their results showed that ICP was significantly lower in patients with POAG compared to non-glaucomatous controls. Reduced ICP was also found in NTG patients with a statistically calculated ICP that was lower than that found in POAG. In contrast, ICP was elevated in patients with ocular hypertension in comparison to no disease controls (Fig. 2) (18, 37). Subsequently, the TLPG was found to be higher in patients with glaucoma (18). Correlation between ICP and other established risk factors for glaucoma was also investigated. In separate publications they reported that starting in the sixth decade of life, there is a “sustained and significant reduction” of ICP with age. The age at which ICP began to decrease also coincided with the age when prevalence of glaucoma increases (51). Additionally, body mass index (BMI), which has been reported to be a risk factor for POAG (52), was found to have a positive linear correlation with ICP (53). This implied that patients with higher BMI may be at a lower risk of developing glaucoma owing to a higher ICP. This concept was supported by a different study showing that females with a higher BMI had a lower risk of developing POAG (54). Although the authors suspected the endocrine role of adipose tissues behind this effect, Fleishman et al. believed that this was due to a concomitant increase in ICP in patients with higher BMI. Arguably, the elevated ICP in these patients can provide a compensating counter pressure on the lamina cribrosa and lower the propensity of the optic disc to bow posteriorly in response to a higher IOP (55).

Shortly after the publication of the retrospective studies, several prospective studies showed that in NTG patients, ICP was abnormally low, causing a higher than normal TLPG. This pressure was even lower than that of patients with high-pressure glaucoma while the latter was significantly lower compared to normal control (56). These results were corroborated by animal studies performed on non-human primates. By lowering the ICP with the help of a shunt, glaucoma like pathology was observed in 50% of the experimental animals. These changes included reduced thickness of the retinal nerve fiber layer, reduction in area and volume of the neuroretinal rim, and a significant increase in cup-disk ratios (57). When ICP was measured non-invasively using a specialized transcranial Doppler, it was found that ICP was 2-3 mmHg lower in patients with NTG and open angle glaucoma when compared to healthy controls (58).

## ICP-IOP-TLPG AND THE CSF THEORY – WHAT DO THE DATA SIGNIFY?

Based on convincing experimental and clinical reports, it is evident that ICP is a significant risk factor for glaucoma. Low intracranial pressure may be considered as the “tipping point” in scientific knowledge regarding glaucomatous pathology (59). Health of the optic nerve head along with surrounding neuronal tissue is maintained by a homeostatic pressure difference between a posteriorly directed IOP and an anteriorly directed ICP. These two pressure components are separated by the thin lamina cribrosa and give rise to the TLPG. Owing to the biomechanical nature of the optic nerve head, TLPG may be the single most important pressure related parameter for the development and progression of glaucoma (33, 60). This was also shown in a study where a reversal of the TLPG following Valsalva maneuver was found to affect the three-dimensional optic nerve head morphology and other parameters associated with glaucoma like pathologies (61). Therefore, as the retrospective and prospective studies show, an increase in IOP (as found in many glaucoma patients) will have the same effect on TLPG as a lowered ICP. In a recent case report, Chen et al. described how NTG of a patient worsened after ICP reduction following surgical implantation of a ventriculoperitoneal shunt (62).

Based on the CSF theory of glaucoma, researchers have now found evidence linking glaucoma and Alzheimer’s disease (AD). Studies show that depending on race and ethnicity, there could be as high as a 5-fold increase in the prevalence of glaucoma in AD patients when compared to the average population (63, 64). Although in a small subset of patients with early AD, ICP was found to be elevated; the same study reported a substantially larger population with more advanced AD to have a significantly lower ICP, which subsequently gave rise to an abnormally high TLPG (65-67). In a recent retrospective study, Fleishman et al. have also reported a similar significantly lowered ICP in AD patients in the age group of 40-69 years (68). These data support the hypothesis that low ICP and a high TLPG found in AD patients could be the reason behind comorbidity of glaucoma and AD (69). 

Mounting evidence suggests that there may be a cross-talk between IOP and ICP, with changes in one compensated by the other at least within a biological range. In monkeys, experimentally increased ICP was found to elevate IOP within a few minutes of induction. This phenomenon continued as long as the pressure in the brain was below blood pressure. ICP increases beyond blood pressure did not elicit any changes in the IOP (70). In a separate study on a population of 50 patients, Sajjadi et al. found a high correlation between IOP and ICP (r=0.995) (71). Similarly, Lashutka et al. have shown that high IOP measured with a handheld tonometer is a useful indicator of elevated ICP especially in patients with intracranial lesions and without glaucoma (72). Indeed, ICP is an important consideration in the diagnosis of various eye diseases (73). The relationship between ICP and IOP may be explained by a recent report by Samuels et al. where the authors have shown that both ICP and IOP are physiologically regulated by the same group of neurons in the dorsomedial and perifornical hypothalamus (74). Nevertheless, the relationship between ICP and IOP is not well understood, and some studies have also shown a lack of correlation between ICP and IOP (75, 76). Although changes in IOP might still be useful in providing some evidence to changes in ICP, the inherent variability of the correlation between these two parameters reduces its clinical significance (77). It is also possible that the relationship between IOP and ICP breaks down under pathological conditions, when one of the two changes outside the normal range (29). 

The reverse effect of the CSF theory can be seen in clinical conditions like idiopathic intracranial hypertension (IIH), pseudotumor cerebri and ocular hypotony. All of these conditions cause an unbalanced anteriorly directed force either due to an elevated ICP and normal IOP (IIH, pseudotumor cerebri) or a normal ICP and low IOP (ocular hypotony). The net result is a negative cupping effect or swelling of the optic disk – a condition called papilledema, with glaucoma like loss of retinal neurons (17, 26, 33, 78). Several case reports have shown development of papilledema in patients with both IIH and ocular hypertension, who were treated with IOP-lowering surgery (79-81). This indicates that patients with IIH may tend to have ocular hypertension to counterbalance the increased ICP, thereby maintaining a homeostatic TLPG (78). Based on this, it has been proposed that a clinically increased IOP may be a treatment option for patients with papilledema (78, 82, 3).

The mechanical effect of the ICP component on the lamina cribrosa is further underscored by reports of “reversal of cupping” in patients whose IOP has been lowered below normal levels for 5 years (84, 85). Obviously, this reversal does not improve the visual field since retinal neurons once lost cannot regenerate, but it does indicate the importance of ICP on maintenance of optic nerve health.

## ANIMAL MODELS TO STUDY THE ROLE OF ICP IN GLAUCOMA

**Figure 3:**
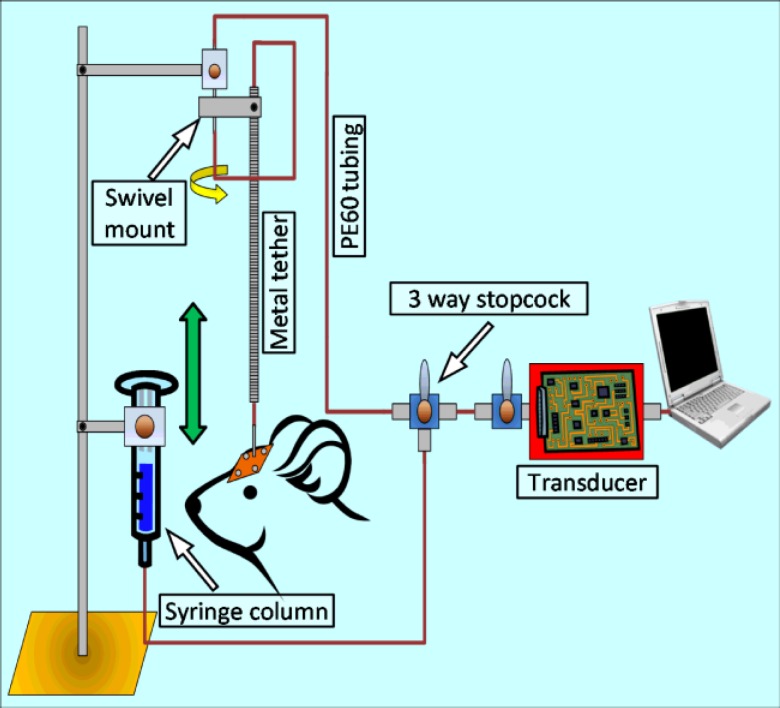
Schematic representation of the rat IVC model.

Despite a large body of evidence in favor of the CSF theory, there still exist doubts as to the contribution of ICP in the pathogenesis of glaucoma (86). This is mainly due to the absence of a viable animal model where the relationship between ICP, IOP and relevant optic neuronal tissues can be studied in a controlled environment (59). The dog model of Morgan et al. was key to our understanding of the anatomic and physiologic significance of ICP and IOP. This model, through experimental alteration of ICP and IOP, also elaborated the mechanical role of TLPG in causing optic disk movements (20, 23, 87). These data highlight the susceptibility of optic neuronal components under altered IOP and/or ICP (7). However, evaluating glaucoma like neuronal pathologies in the eye following chronic changes in ICP/TLPG was beyond the scope of this model. To overcome this, our laboratory has recently designed a novel rat intraventricular cannula (IVC) model, which allows investigators to manually increase or decrease the ICP over sustained periods of time (Fig. 3) (88).

ICP was monitored by connecting a pressure transducer to a surgically implanted cannula in the left lateral ventricle. ICP can be manually raised or lowered in a controlled fashion via elevation or declination of an artificial CSF containing reservoir connected in parallel to the primary setup. Reprinted from Roy Chowdhury et al. (88).

Using the rat IVC model, long-term lowering of ICP resulted in a significant reduction of retinal ganglion cell and optic nerve axon density (Roy Chowdhury U et al, IOVS 2016;56:ARVO-E abstract 4131). This model may be instrumental in bridging the current knowledge gap regarding ICP and glaucoma. Recently, Yang et al. have described a monkey model where CSF was lowered by implantation of a lumbar-intraperitoneal CSF shunt. Health of the optic tissues was monitored using optical coherence tomography. The authors reported that two of the four experimental animals showed characteristic signs of glaucomatous pathology after ICP was lowered by at least 5 mmHg (57). Although the monkey model of Yang and colleagues is extremely relevant and an elegant setup (57), the IVC model is comparatively inexpensive and would require less upkeep and operational budget. Nevertheless, both these animal models will help us better understand the role of ICP and TLPG in glaucomatous pathogenesis.

## SUMMARY AND CONCLUDING REMARKS

Cupping of the optic nerve head and the lamina cribrosa is a characteristic pathological manifestation of glaucoma. Under homeostatic conditions, the lamina cribrosa is subjected to a posteriorly directed pressure of roughly 4 mmHg (17). This pressure difference arises out of the posteriorly directed IOP and the anteriorly directed ICP which creates a pressure gradient across the lamina cribrosa, known as the TLPG. IOP and ICP are anatomically and physiologically interlinked pressure components and changes in one may be reflected by changes in the other within a biological range. Under pathological conditions like glaucoma, a low ICP in the presence or absence of a high IOP will cause an increasingly unbalanced posteriorly directed force on the lamina cribrosa. The thickness of the lamina cribrosa and the resulting elastic resilience of the surrounding sclera also play a significant role in maintaining the homeostatic condition around the optic nerve head.

Considering all retrospective, prospective and experimental evidences, ICP should be regarded as an important factor in the progression of glaucoma. Studies in animal models like the rat IVC model will enable the determination of the relationship between ICP, IOP and glaucoma. Standardizing non-invasive and surrogate techniques to identify ICP values will be of particular importance, since current methods are highly invasive and dangerous (59, 89, 90). Notwithstanding the technical difficulties, the current data should help educate the clinical and research communities about the importance of ICP in diagnosis and treatment of glaucoma. Hopefully, the knowledgeable physician will now look for signs of glaucoma in patients with subtle neurological symptoms of low ICP like postural headaches and sixth nerve weakness (3). 
